# PBL teaching in ultrasonography resident standardization training in the COVID-19 pandemic

**DOI:** 10.1186/s12909-022-03555-9

**Published:** 2022-06-30

**Authors:** Zi-mei Lin, Yu-rong Hong, Chun-mei Liu, Zhi-yan Luo, Ying Zhang, Xiao-jie Xie, Pin-tong Huang

**Affiliations:** 1 Department of Ultrasound in Medicine, The Second Affiliated Hospital Zhejiang University School of Medicine, No. 88, Jie Fang Road, Hangzhou, 310009 Zhe Jiang Province China; 2 Department of Teaching, The Second Affiliated Hospital Zhejiang University School of Medicine, No. 88, Jie Fang Road, Hangzhou, 310009 Zhe Jiang Province China

**Keywords:** PBL, LBL, Ultrasound, Residents, COVID-19

## Abstract

**Objective:**

To study the effect of the problem-based learning (PBL) method in ultrasonography (US) resident standardization training during the COVID-19 pandemic.

**Methods:**

Fifty residents were divided into two groups to participate in a 30-day US training program. The residents in the observation group underwent PBL combined with the lecture-based learning (LBL) method, while the residents in the control group experienced the LBL method alone, with 25 residents in each group. A basic theoretical test, practical examination, and questionnaire were used to evaluate the teaching effect of the PBL + LBL method and the LBL method alone.

**Results:**

The basic theoretical pretest score of the observation group was not significantly different from that of the control group. However, the posttest theoretical score and practical score were significantly higher in the observation group than in the control group (*P* < 0.01). The results of the questionnaire showed that the resident satisfaction level in the observation group with PBL combined with the LBL method was 96%, which was significantly higher than that of the control group with the LBL method alone (80%) (*P* < 0.05).

**Conclusion:**

The combination of PBL with the LBL method has obvious advantages over the LBL method alone in regard to the training of US residents during the COVID-19 pandemic.

**Supplementary Information:**

The online version contains supplementary material available at 10.1186/s12909-022-03555-9.

## Introduction

The World Health Organization (WHO) declared COVID-19 a pandemic, and this pandemic has struck most countries across the globe [1]. The effects of COVID-19 range from political, economic, social, and health systems to education and have brought radical changes to lives all over the world. Social distancing and restrictive movement policies have markedly disrupted traditional educational practices. Due to the global COVID-19 pandemic, many clinical and face-to-face teaching methods have been suspended [2–4]. Health professional education institutions have suffered the most interference, i.e., significantly more than other educational institutions [5]. This result has been mainly caused by the inherent nature of teaching and learning utilized in such institutions, which depend on contact between the teachers, students, and patients in training venues. The longevity of these changes is indeterminate, which has affected conventional ultrasonography (US) education and training. The COVID-19 pandemic has provided us with an opportunity to pave the way for introducing innovative teaching methods. Almost all educational institutions have chosen to implement online methods to maintain the continuity of education across academia [6–8]. Some institutions started with preparing PowerPoint presentations with voiceovers and sharing them with the residents via different applications. Platforms such as learning management systems (LMSs) have also been used to upload various learning materials to encourage students in learning activities [9–11]. US residency programs should continue to adapt as the pandemic situation unfolds over time. The current pandemic may serve as a catalyzer for the integration of technology and simulating diagnosis in resident education, which are factors that are likely to become permanent part of the future of US education. Various electronic resources and strategies have been used to sustain academics during this pandemic. Lecture-based learning (LBL) is a traditional teaching method based on teachers’ courses. It is challenging for radiologists to continuously engage residents online during the pandemic due to issues such as attention spans, poor dynamic video quality, and internet issues [12]. Problem-based learning (PBL) is a problem-oriented teaching method that guides residents to actively find and analyze problems and to acquire the skill of autonomous learning, which is quite different from traditional teaching methods [13–15]. We tried to evaluate the effectiveness of PBL in US resident standardization training during the COVID-19 pandemic.

## Methods

The Second Affiliated Hospital ZheJiang University School of Medicine (SAHZJU) is an academic center that is home to a 3-year residency program training and a tertiary care facility. US residents at our center who were in years 2–3 of the training program both before and during the pandemic, i.e., from September 2018 to September 2021, were prospectively invited to participate in this study. The study received institutional review board approval by the committee for clinical investigations at our center (NO. 2021–1088). These US residents were licensed and had at least 1 year of experience in our department. First-year residents who had just joined the training program were not included. Then one month was randomly selected with computer, the US residents trained in our department this month were selected as the case group (PBL combined with the LBL method was used), and other US residents trained in other departments were the control group (the LBL method alone was used), with 25 residents in each group. Before the start of the study, each resident completed a pretest to determine the baseline levels. Both groups were taught identical teaching content using a standardized syllabus. The training course lasted for 30 days and was divided into theory training and hands-on instructions. Lectures and talks, including liver, gallbladder, pancreatic, and spleen theoretical courses (8 classes, 1 h per class) that were normally delivered online using the teleconferencing program Ding-talk (Ding-talk Communications, Alibaba, China), were replaced by online video archives. The hands-on ultrasound skills instructions, including clinical application courses for the liver, gallbladder, pancreas, and spleen (4 classes, 1 h each), were replaced by online demonstrations. In this course, residents were introduced to the general principles of ultrasound, equipment operation, and workflow understanding; comparisons of normal anatomy and physiology to pathological features and the differential diagnoses of such findings were also introduced.

The residents in the control group, i.e., the LBL method alone, were taught in the traditional method (including all the lectures of liver, gallbladder, pancreatic, and spleen) online. The residents in the observation group were provided with information related to the PBL method, in addition to the content used in the LBL method. The PBL method was used to provide timely answers and guidance for the problems encountered by residents. Meanwhile, teachers with rich clinical and teaching experience (more than 3 years) were arranged to organize discussions, which residents were timely notified of and organized to participate in.

At the end of the study, the residents completed a written posttest. Both the pre- and posttests covered physics, a basic examination, and liver, gallbladder, pancreatic, and spleen sonography; they also used a multiple-choice format. The questions were divided into three categories, namely, knowledge-based questions (35 questions), interpretation questions (25 questions), and clinical decision-making questions (40 questions), for both the pre- and posttests. In addition to the theory examination, at the end of the study, the residents needed to undergo a proctored practical test in which they were graded on their ability to perform an US examination on volunteer patients using a revised version of the Objective Structured Assessment of Ultrasound Skills (OSAUS), which allows the independent and unbiased assessment of ten aspects of ultrasonographic skills and knowledge of the candidate (1. information check, 2. indication, 3. equipment, 4. image optimization, 5. systematic examination, 6. image interpretation, 7. image documentation, 8. management, 9. privacy protection, 10. humanistic solicitude). The maximum score of the assessment was 100, and a passing score was defined as 80.

Focusing on the investigation of satisfaction with teaching methods, the teaching method effect was evaluated through a feedback questionnaire survey. The questionnaire was designed by a qualified radiologist after consulting a psychologist, and it was not validated. The degree of satisfaction with the teaching method included high-level satisfaction (80–100 points), general satisfaction (60–80 points) and dissatisfaction (less than 60 points). Then, the satisfaction degree was calculated according to the following formula: satisfaction degree = satisfaction rate + general rate.

### Statistical processing

SPSS 23.0 software was used, and the Kolmogorov–Smirnov test was used to judge whether the data presented a normal distribution. Measurement data are expressed as the mean ± standard deviation, and a Group t test was used for intergroup comparisons. Scores on pretests and posttests were compared using a Wilcoxon signed-rank test to assess individual differences (paired analysis). *P* < 0.05 was considered a statistically significant difference.

## Results

A total of 3 Ph.D. residents, 21 master residents, and 26 bachelors residents participated in this study. In the control group, there were 7 male residents and 18 female residents, who were aged from 24 to 32 years, with an average of 26.7 ± 2.1 years. There were 3 male residents and 22 female residents in the observation group, who were aged from 25 to 31 years, with an average of 27.9 ± 2.1 years. There was no significant difference in age between the two groups. Significant difference was found in sex and degree between the two groups (Table 1).

**Table 1:** Baseline demographic data

Demographic	Control group	Observation group	*P* value
Sex (M/F)	7/18	3/22	< 0.01
Median age (Years)	26.7 ± 2.1	27.9 ± 2.1	> 0.05
Degree (PhD/Master/Bachelor)	1/12/12	2/9/14	< 0.01

### Comparison of academic performance

All 50 residents completed the training. The median pretest score was 75 (range 66–91), compared to a median post test score of 86 (range 78–97), *P* < 0.01. There was a significant increase in the scores related to US theory and interpretation questions from the pretest to posttest.

The residents in the control group had a median pretest score of 76 and a median post test score of 83. The residents in the observation group had a median pretest score of 75 and a median post test score of 89. There was no significant difference in the pretest score between the control group and the observation group (*P* = 0.12). The posttest score of the observation group was significantly higher than that of the control group (*P* = 0.02). The practical test score of the observation group was significantly higher than that of the control group (91 vs. 85, *P* < 0.01) (Table 2).

**Table 2:** Pre-test and Post-test Scores

Residents	Pre-test Score	Post-test Score	Practical-test score
Observation group	75	89	91
Control group	76	83	85
*P* value	0.12	0.02	< 0.01

### Survey on the satisfaction of residents with different teaching methods

Comparing the satisfaction level of the residents in the two groups, we found that the overall satisfaction level of the observation group was significantly higher than that of the control group (Table 3).

**Table 3:** Satisfaction of Residents

Residents	Satisfaction	General	Dissatisfaction	Satisfaction Degree	*P* value
Observation group	18	6	1	96%	< 0.05
Control group	12	8	5	80%	

## Discussion

According to the results of a large meta-analysis of skill decay and retention literature, the nonuse of procedural skills may cause skill decay [16]. This suggestion has created concerns that some residents may have received inadequate training during the COVID-19 pandemic. Due to the complexity of US, the wide range of knowledge involved, and the fact that a logical mind is difficult to master, it is difficult to achieve educational goals using the traditional LBL method online alone. Therefore, this study aimed to discuss the application of the LBL and PBL methods in the training of US residents in the COVID-19 pandemic.

Due to the possibility of outbreaks among residents, most clinical teaching has either been suspended or converted into online teaching methods based on the concern for infection control [9, 17, 18]. Online education under the COVID-19 pandemic has become an inevitability of education reform and development [19–21]. Online lectures have been able to break through the time and space limitations for US-related teaching during the pandemic [20, 21]. Many technologies and softwares have been developed to make up for the suspension of clinical teaching. Previous research has shown that distance learning is noninferior, if not superior, to other methods of learning in terms of operating skills and anatomy teaching [22, 23]. However, the traditional LBL online method is mainly based on radiologists’ medical imaging lessons so that the students can obtain a systematic state of the etiology, pathology, clinical features, imaging features, and course of disease [24]. This content is easy to manipulate in the LBL method, while the participation level of the residents is often lower, which restricts the development of the residents’ minds. On this basis, the PBL method has changed the general method of theory acquisition in medical education, stimulated residents’ learning motivation with radiology problems, and guided residents to grasp the key points. Thus, PBL-based medical learning has begun to take root and grow in many medical schools [25, 26]. The introduction of PBL into teaching is quite different from the traditional LBL method. The comparison between the PBL method and the LBL method is similar to a comparison between active learning and passive learning. Therefore, it is reasonable for residents who are using the PBL method to understand and master US theory better than those who are using the LBL method alone. The results of this study showed that the knowledge level increased in both groups after the study, while the theoretical score outcome of the PBL + LBL group was significantly higher than that of the control group. Residents using the PBL method have tended to place more focus on resource utilization, such as online sources, in the pandemic era. However, those using the traditional LBL method alone have placed more focus on the resources provided by the radiologists.

US is a comprehensive and practical subject with the characteristics of strong operability and professionalism [27, 28], which means that the subject is very suited to the PBL method regarding helping residents better analyze, infer, synthesize, evaluate, think about and inquire into how US theory applies in the clinic setting. There was significant difference in degree between the two groups, while no significant difference was found in the pretest score between the groups. However, the results of this study showed that the group using the PBL method had higher practical scores compared to those in the control group (LBL method). Previous studies have shown that students who learn through the PBL method are more likely to solve problems spontaneously in the future than are those who participate in a traditional learning method alone [29, 30]. In the traditional LBL method, residents often lack the comprehensive ability to solve complex affairs. This affects the students’ strengthening process of transitioning from theory to practice and then back to theory, which might not cultivate their practical operation ability and clinical skills. The principle of PBL is to provide residents with a task or challenge as a source for learning, encourage them to express their ideas and thoughts based on prior tasks or challenges, and simulate situations that will occur in clinical practice in the future [31–33]. Based on a critical analysis of the issue, residents participated in the course, mastered what they were taught by radiologist instructors, and developed the required questioning and clinical thought skills.

The questionnaire survey in our study showed that residents' satisfaction level with US courses was significantly improved after using the combined learning method. The traditional LBL method has difficulty mobilizing residents' learning enthusiasm. However, the residents’ learning appeared more ‘live’ and ‘active’ when using the PBL method, which might contribute to student satisfaction, motivation and critical thinking levels [31, 34, 35]. Residents assumed responsibility for their own learning. The radiologist instructors acted as guides and facilitators instead of only providing information. Residents became self-directed and collaborative in their studies. Another important advantage of PBL was that most of the PBL cases were based on organ systems, which suited the subspecialization of US.

The sample size was small in the current study, and it was a single-center study. US residents at our center who were in years 2–3 of the training program both before and during the pandemic participated. Therefore, caution should be exercised when analyzing the results, and residents of other majors should also be included in research to verify the effect of the PBL method in the future. In addition, the questionnaire was nonvalidated and designed by a qualified radiologist after consulting a psychologist. Another limitation was that the study did not compare the perception of residents of different training years, which was due to having insufficient samples from individual training years to provide us with valid comparative results. A fourth limitation of the PBL method was the insufficient time allocated to cover the basic theory of US feature interpretation compared with that used in the traditional LBL method. It might be difficult to incorporate basic concepts into a PBL course that is based on clinical cases, with the risks of ignoring theory. That is why we applied the combination of the PBL and LBL methods in the observation group rather than use the PBL method alone.

In summary, the combination of PBL and the LBL teaching method can significantly improve residents' theoretical and practical performances and is popular with residents in the era of COVID-19. Thus, the PBL method of distance learning should be further explored.

## Supplementary Information

**Additional file 1.**

## Data Availability

The datasets generated and/or analysed during the current study are not publicly available due to limitations of ethical approval involving the participants data and anonymity but are available from the corresponding author on reasonable request.
