# Supplementary material for: PBL teaching in ultrasonography resident standardization training in the COVID-19 pandemic
Source: BMC Med Educ. 2022 Jun 30;22:512. doi: 10.1186/s12909-022-03555-9 (PMC9245401; doi:10.1186/s12909-022-03555-9)
Supplement: Supplementary file 1 — Additional file 1. [file 12909_2022_3555_MOESM1_ESM.docx]

1. Questionnaire
2. What's your gender?

2. How old are you?

3. What is your educational background?

4. What's your major in college？

5. How many case discussions have you participated in (this month)?

6. How many case discussions have you participated in (from the start)?

7. Do you think case discussion is helpful for mastering knowledge?

8. What is the effect of online case discussion?

9. What is the score of satisfaction with online teaching mode? (0-100 points)

10．Do you have any other suggestions for the current US training in this era?

1. Some of the tests
2. The liver was divided into 8 segments by Couinaud segmentation method, in which the caudate lobe was?

A.Ⅰ段

B.Ⅱ段

C.Ⅲ段

D.Ⅴ段

E.Ⅷ段

1. The direct signs of extrahepatic cholangiocarcinoma do not include:

A. The distal end of the dilated bile duct shows a papillary soft tissue mass

B. The proximal bile duct of obstruction was significantly dilated

C. The lumen of the dilated bile duct gradually became thin and narrow in the shape of rat tail, and the local tube wall was significantly thickened

D. Irregular thickening and rigidity of bile duct wall

E. Sudden truncation or occlusion of bile duct

3. A patient with acute abdominal pain. US found multiple stones in the gallbladder, diffuse enlargement of the pancreas, weakened and uneven echo, thin and narrow splenic vein, weak vocal cords around the pancreas, intestinal dilatation, and a large amount of effusion with poor sound transmission in the abdominal cavity. The most likely diagnosis is

A. Edematous pancreatitis

B. Acute cholecystitis

C. Intestinal obstruction

D. Chronic pancreatitis

E. Hemorrhagic necrotizing pancreatitis

4. Male, 56 years old, developed epigastric pain without obvious inducement 2 months ago, which was not severe and tolerable.

CT of local hospital (February 16, 2019) indicates: chronic liver disease, large spleen, cystic space occupying in V segment of liver, considering infectivity pathological changes. The effect of symptomatic treatment is poor. Come to our hospital for treatment.

Physical examination: V / s 126 / 62, R 19 HR 86, t 37 ° C, upper abdominal tenderness (+) Murphy's sign (-)

Laboratory inspection (February 19, 2019):

- CBC: WBC 10.1 10^9/L Hb 125g/L
- LFT: Dbil 5.6 umol/L ALT 61 U/L, AST 63 U/L; AKP 176 U/L.
- Tumor markers: SCCA 1.8 ng/ml. CA199 40.1kU/L
- HBV marker: HBsAg +, HBcAb+


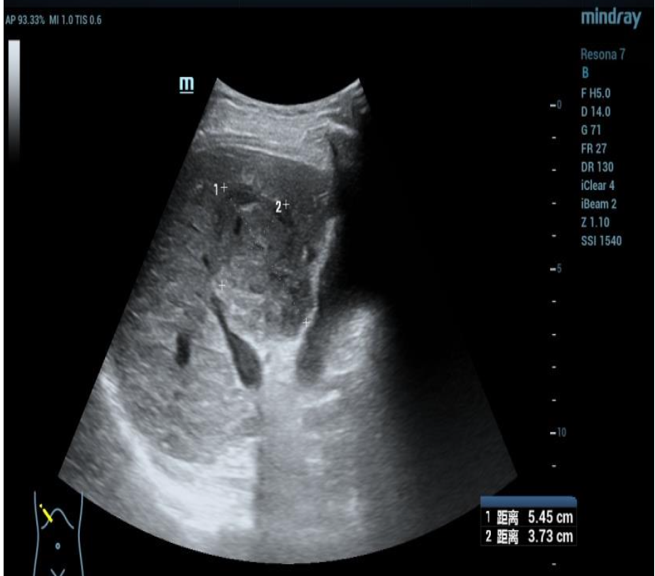

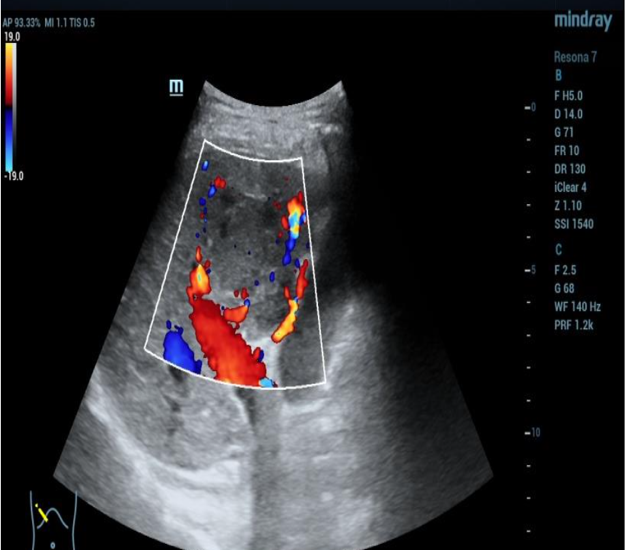


1. According to the information above, what’s diagnosis?

A. hepatic abscess

B. HCC

C. hepatic cyst with infection

D. hepatic hemangioma

1. What are the next recommendations？

A. surgery

B. CT

C. CEUS

D. MRI

E. CNB
